# Effects of selected lactobacilli on the functional properties and stability of gluten-free sourdough bread

**DOI:** 10.1007/s00217-017-3020-1

**Published:** 2017-12-13

**Authors:** Denisse Bender, Vera Fraberger, Palma Szepasvári, Stefano D’Amico, S. Tömösközi, G. Cavazzi, H. Jäger, Konrad J. Domig, Regine Schoenlechner

**Affiliations:** 1 Department of Food Science and Technology, BOKU-University of Natural Resources and Life Sciences Vienna, Muthgasse 18, 1190 Vienna, Austria; 2 Department of Applied Biotechnology and Food Science, Budapest University of Technology and Economics, Müegyetem rkp.3, Budapest, 1111 Hungary; 3 Department of Food and Nutritional Sciences, University of Milan, Via Celoria 2, 20133 Milan, Italy

**Keywords:** Gluten-free bread, Sourdough, Lactobacilli, Back-slopping

## Abstract

The aim of this investigation was to determine the influence of seven different *Lactobacillus* spp. (Lb.) strains compared with a commercial starter culture (CS) on the functional properties of gluten-free (GF) sourdough-breads. The sourdough stability of selected strains was also evaluated upon back-slopping. Results showed that the bread properties were greatly affected by the Lb. strains. Millet breads achieved lower specific volumes (1.80–2.19 cm^3^/g), higher crumb firmness (19.01–42.19 N) and lower relative elasticities (21.5–43.4%) than buckwheat breads. Compared with the CS, *Lactobacillus pentosus* and *Lb. hammesii* positively influenced the crumb firmness of buckwheat and millet breads, respectively, while *Lb. paralimentarius* enhanced this property in both breads. Only one of the two *Lactobacillus sanfranciscencis* strains was able to improve all functional properties in both GF breads. Back-slopping of the sourdoughs revealed stable properties in case of buckwheat, while maturity of the millet sourdough could not be reached. These observations were supported by the microbial count, metabolite production and carbohydrate consumption. Mature sourdough significantly improved the crumb firmness and porosity of the GF breads. These results highlighted the importance of selecting the appropriate lactic acid bacteria strains, to maximize the quality of GF bread.

## Introduction

The replacement of gluten in GF bread still represents a great technological challenge since GF batters lack cohesive and elastic structure, which makes achievement of high quality bread properties and industrial handling of batter a great challenge. In the past decade, application of GF ingredients such as hydrocolloids, starch and dairy products were used for the development of GF products. Nowadays, exploiting novel or even ancient processing approaches, such as enzymatic processing, high hydrostatic pressure processing, sourdough technology and extrusion technology has been the main focus. With these methods, the viscoelastic properties of wheat dough can be imitated to some extent, while improving GF batter handling properties [1].

Sourdough is a mixture of water and flour, which is fermented by lactic acid bacteria and yeasts [2] and has been used to improve sensory, textural and nutritional properties of bread. According to some studies [3, 4], not only fresh but freeze–dried sourdough has also been able to achieve these improvements, while shortening GF bread production times. Nonetheless, limited research has been aimed to characterize GF sourdoughs and evaluate their effect on the functional properties of sourdough-bread [5–7]. Previous investigations have stated the importance of developing and designing LAB strain combinations for different substrates since commercial starter cultures are not always suitable as such for GF cereals [7, 8]. Therefore, the first aim of this study was to evaluate the compatibility of individual *Lactobacillus* spp. strains in millet or buckwheat sourdoughs and their individual performance to improve bread quality parameters. Since a reproducible and controlled microbial composition of the sourdough is the key factor to achieve a constant bread quality [9], the second aim of this investigation focused on selecting the best performing bacteria and study the fermentation process as well as the stability of the sourdough microbiota during a 10 day back-slopping period.

## Materials and methods

### Materials

Buckwheat and millet grains were bought from Caj. Strobl Naturmühle GmbH (Linz, Austria) and were ground in a pin mill (Fa. Pallmann Maschinenfabrik, PXL 18, Zweibrücken, Germany) at 12,000 rpm for wholemeal flour production. Chemical composition of millet flour was 15.43 ± 0.07% protein, 4.20 ± 0.01% fat, 4.27 ± 0.09% total dietary fiber and 66.16 ± 1.53% starch, while buckwheat flour was composed of 14.45 ± 0.02% protein, 2.87 ± 0.08% fat, 5.87 ± 0.10% total dietary fiber and 66.29 ± 2.80% starch. These properties were determined according to the official ICC Standards Methods. For baking, instant yeast was donated from Lesaffre Austria AG (Wiener Neudorf, Austria). A commercial gluten-free sourdough starter culture (CS) (Böcker Reinzucht Sauerteig Reis) was bought from Ernst Böcker GmbH & Co. (Minden, Germany). All used chemicals and reagents were of analytical grade and purchased from Sigma–Aldrich (Steinheim, Germany).

### Microorganisms, strains and cultivation conditions

The strain details including the source of isolation of the lactobacilli applied in this study are presented in Table 1. All *Lactobacillus* spp. strains applied were stored at − 80 °C mixed with 20% Glycerol (86%). For cultivation MRS-broth and MRS-agar (Merck, Germany) was used and incubation took place at 30 °C under anaerobic atmosphere (10% CO_2_, 10% H_2_, 80% N_2_).

**Table 1:** Species of lactic acid bacteria used and their source of isolation

Strain code	Genus	Species	Strain	Source of isolation
Lbf	*Lactobacillus*	*fermentum*	LMG 6902^a^	Fermented beets
Lbh	*Lactobacillus*	*hammesii*	DSM 16381^a^	Wheat sourdough
Lbpa	*Lactobacillus*	*paralimentarius*	LMG 19152^a^	Sourdough
Lbpe	*Lactobacillus*	*pentosus*	LMG 10755^a^	Unknown
Lbpl	*Lactobacillus*	*plantarum* subsp. *plantarum*	LMG 6907^a^	Pickled cabbage
Lbs1	*Lactobacillus*	*sanfranciscensis*	LMG 16002^a^	Sourdough
Lbs2	*Lactobacillus*	*sanfranciscensis*	DSM 20663	Sourdough

LMG… Belgian Coordinated Collections of Microorganisms (Gent, Belgium)

DSM…Leibniz-Institute DSMZ-German Collection of Microorganisms and Cell Cultures (Braunschweig, Germany)

^a^Type strain of the species

### Sourdough preparation and gluten-free bread production

Table 2 displays the formulations of millet and buckwheat sourdough and sourdough-bread production. Apart from inoculating sourdough with pure *Lactobacillus* spp. strains, a commercial gluten-free sourdough starter culture (CS) was used for comparative purposes. For preparation of the commercial sourdough, the CS was dissolved in water in a 1:10 ratio, added to the flour and subsequently fermented at 25–27 °C and 85% R.H. for 16–18 h in a fermentation chamber (Model 60/rW, MANZ Backtechnik GmbH, Creglingen, Germany). The resulting sourdough was immediately used for baking.

**Table 2:** Formulation of gluten-free sourdough and sourdough-bread

Ingredients	Buckwheat		Millet	
	Amount (g)	% of total flour weight	Amount (g)	% of total flour weight
Commercial sourdough (inoculated with CS)				
Flour	250.0		250.0	
Water	250.0	100.0	250.0	100.0
SC	25.0	10.0	25.0	10.0
Sourdough inoculated with *Lactobacillus* spp. strains				
Flour	250.0		250.0	
Water	200.0	80.0	250.0	80.0
Lactobacilli suspension (mL)	50.0	20.0	50.0	20.0
Model GF bread batter				
Flour	314.0		314.0	
Sourdough (flour:water ratio = 1:1)	422.0		422.0	
Salt^a^	10.0	1.9	10.0	1.9
Sugar	10.0	1.9	10.0	1.9
Yeast	10.0	1.9	10.0	1.9
Water	314.0	100.0	251.2	88.0

*SC* commercial starter culture

^a^For the breads using the lactobacilli suspension, 0.3 g less salt was added, considering the salt content of the broth

For the inoculation of sourdough with *Lactobacillus* spp. strains, the bacteria were cultivated in 9 mL MRS-broth for 72 h at 30 °C, further transferred into 200 mL fresh MRS-broth and incubated again for 24 h. Afterwards, cell wash was applied to remove residual media components. Then, a resuspension of the cell pellet (approx. 10^7^ cfu/g) was made using 50 mL of 0.8% NaCl-solution, as this amount of lactobacilli is suggested to lead to an increased probability for dominant growth of the selected strains and suppression of the natural microbiota of flour [10]. For sourdough production, the 50 mL microbial suspension was dissolved in 200 g of water and mixed with 250 g of flour. Afterwards, the sourdough was fermented at 25–27 °C and 85% R.H. for 16–18 h and immediately used for baking.

For model GF bread baking, a simplified GF recipe was selected (Table 2). For salt addition of all formulations inoculated with the purified *Lactobacillus* spp. strains, its content in the broth was considered. Water addition was optimized in pre-trials according to the raw materials (data not shown). No isolated protein, emulsifier or hydrocolloid was added, to exclude any additional effects. The GF batter was made by mixing all dry ingredients with a laboratory dough mixer (Bär Varimixer RN10 VL-2, Wodschow & Co., Denmark) for 1 min. Then, sourdough was added and water was slowly poured into the bowl and mixed at step 2 for 6 min. The batter was rested 10 min at 30 °C and 85% R.H. in a fermenter (Model 60/rW, MANZ Backtechnik GmbH, Creglingen, Germany). Afterwards, two portions of batter (400 g) were accurately weighed into baking tins and consecutively fermented at 30 °C and 85% R.H. for 40 min. Breads were baked at 180 °C for 35 min in case of millet and 40 min for buckwheat (Model 60/rW, MANZ Backtechnik GmbH, Creglingen, Germany). Loaves were kept at 20 °C and 50% R.H. for 24 h before being evaluated. Baking trials were performed in duplicate measurements, resulting in four loaves for each formulation.

### Back-slopping experiments

Based on the individual performance of the *Lactobacillus* spp. strains, a combination of four strains was chosen to study the performance of the microorganisms and the sourdough fermentation process during a consecutive 10-day refreshment stage. Sourdough was prepared by diluting 50 mL of the physiological saline solution containing at least 10^7^ cfu of each Lb. strain/g in 200 mL of water and mixing it with 250 g of flour. This procedure was performed in duplicate trials for buckwheat (B1, B2) and millet (M1, M2). Each batter was fermented at 25–27 °C and 85% R.H. for 24 h. A 20 g sample was taken for analysis, while 25 g were taken for refreshing which was done by diluting 25 g of this sourdough in 250 g of water, mixed with 250 g of flour and fermented at 25–27 °C and 85% R.H. for 24 h. Refreshments were repeated every 24 h for 10 days, taking 20 g of sample on the days 0, 1, 2, 5 and 10 before back-slopping for chemical analysis of the sourdough and quantification and identification of lactic acid bacteria. During this period, pH and total titratable acidity (TTA) of the sourdoughs were analyzed daily. Baking tests were performed using each of the characterized sourdoughs (B1, B2, M1 and M2) at day 1 and 10 and bread properties were evaluated.

### TTA, pH and organic acids measurement

The pH was determined using a pH-meter Testo 205 (Testo SE & Co. KGaA, Lenzkirch, Deutschland). TTA was expressed as the amount (in mL) of 0.1 M NaOH needed to achieve a final pH of 8.5 of the sample. The measurement was made following the ICC Standard method no. 145 [11]. Organic acids, ethanol and carbohydrates were determined as described by Jekle et al. [5] with some modifications. A 2.0 ± 0.01 g portion of sample was added to 20 g of degassed 5 mM H_2_SO_4_, homogenized for 1 min and centrifuged at 4000 rpm for 5 min. Subsequently, the supernatant was recovered and the pH was adjusted to 4.0. A 2 mL aliquot from the sample was centrifuged at 14,000 rpm for 10 min and filtered through a 0.2 µm filter (25 mm filter, polyamide membrane, VWR International GmbH, Darmstadt, Germany). Samples were analyzed using an IEC dual analysis system ICS-5000 (Dionex, USA), equipped with an AMINEX HPX-87H analytical column (300 mm × 7.8 mm). Detection of carbohydrates and ethanol was carried out with a Shodex RI 101 refractive index detector (Showa Denko K.K., Kawasaki, Japan) while organic acids were identified using an UV–vis detector (UDV 170U, Dionex, USA) set at 210 nm. The system was maintained at 50 °C using 5 mM H_2_SO_4_ as a mobile phase with a flow rate of 0.6 mL/min at an isocratic gradient.

### Bread quality determination

Specific volume of the loaves was determined using the rapeseeds replacement method following the AACC Approved Method 55-50.01 [12]. Specific volume (cm^3^/g) was calculated as the ratio of the volume (cm^3^) and the mass of the bread (g). Measurements were carried out in duplicates, obtaining eight values for each formulation.

Crumb firmness and relative elasticity were measured as described by Phimolsiripol et al. [13] with some modifications. A compression test was carried out using a Texture Analyzer (Model TA-XT2i, Stable Microsystems™ Co., Godalming, UK) equipped with a 5 kg load cell and an SMS 100 mm diameter compression probe (P/100). Rectangular samples of 3 × 3 × 2 cm (*L* × *W* × *H*) were cut from the center of the loaves, removing top and bottom crust and subjected to an uniaxial compression of 25% strain at 0.5 mm/s speed. Pre-test speed and post-test speeds were 5 mm and 10.0 mm/s, respectively. After compression, a relaxation time of 120 s was applied. The crumb firmness represented the maximum force (*F*_max_) needed to deform each cube. The relative elasticity (REL) in percent was calculated by dividing the residual force (*F*_res_) at the end of the relaxation time by the maximum force (*F*_max_) and multiplied by a factor of 100. Duplicate measurements of each loaf were performed, obtaining eight values for each formulation.

### Quantification and isolation of LAB

To examine the microbiological count, 10 g of sourdough was diluted 1:10 with peptone water and homogenized for 30 s at 230 rpm in a Stomacher^®^ 400 Circulator (Seward, UK). LAB were estimated and isolated by plating on MRS-agar supplemented with 10 mg/L cycloheximide (Carl Roth GmbH, Karlsruhe, Germany). Pure cultures were selected due to morphological differences further grown in MRS-broth and after an incubation time of 72 h cells were gained by centrifugation.

DNA for PCR reactions was extracted using a DNA-extraction kit (peqGOLD Bacterial DNA-Kit, VWR, International GmbH, Germany). Afterwards, 16S rDNA PCR was performed on a Mastercylcer nexus (Eppendorf, Austria) according to Brändle et al. [14].

PCR products were further send for sequencing to Eurofins Genomics GmBH (Germany) and received sequences were analysed using the Basic Local Alignment Search Tool BLASTN (https://blast.ncbi.nlm.nih.gov/).

### Data analysis

Statistical analyses were performed using STATGRAPHICS Centurion XVII, version 17.1.04 (Statpoint Technologies, Inc., Warrenton, VA, USA). Results of all parameters are expressed as mean ± standard deviation. One way ANOVA (analysis of variance with *α* = 0.05) Games–Howell and Fishers least significance tests were used to determine statistical significant differences between formulations. Significant differences were indicated by different letters in the rows when *p* value was lower or equal to 0.05.

## Results and discussion

### Selection of *Lactobacillus* spp. strains for buckwheat and millet sourdough-bread production

To select the appropriate starter cultures for the production of millet and buckwheat sourdough-bread, the effect of seven *Lactobacillus* spp. strains on the functional bread properties was determined and compared with the performance of a standard commercial gluten-free starter culture. Moreover, since the pH itself can influence the texture and stability of the batters, the pH of the sourdough and bread batters before and after fermentation was monitored. TTA was also measured to provide complementary information about metabolic activity and growth of the lactobacilli during fermentation. Table 3 displays the influence of different *Lactobacillus* spp. strains on the chemical and technological properties of millet and buckwheat sourdough and sourdough-breads.

**Table 3:** Influence of different *Lactobacillus* spp. strains on the chemical and technological properties of millet and buckwheat sourdough and sourdough-breads

LAB strain	Buckwheat			Millet		
	Specific volume (cm^3^/g)	Firmness (*N*)	REL (%)	Specific volume (cm^3^/g)	Firmness (*N*)	REL (%)
Lbf	1.93 ± 0.07^a^	9.46 ± 2.29^b,c^	57.41 ± 2.81^c,d^	1.99 ± 0.05^c^	41.91 ± 3.69^d^	33.5 ± 2.08^c^
Lbh	2.18 ± 0.06^b^	18.43 ± 1.49^d^	52.63 ± 3.89^a,b^	1.93 ± 0.02^b,c^	20.53 ± 1.57^a^	32.0 ± 3.84^d,e^
Lbpa	2.01 ± 0.04^a^	8.81 ± 0.32^b^	56.84 ± 1.21^c,d^	1.82 ± 0.05^a^	30.25 ± 4.85^b^	21.5 ± 1.72^a^
Lbpe	2.14 ± 0.07^b^	11.12 ± 0.44^c^	59.37 ± 2.75^c,d^	1.87 ± 0.05^a,b^	34.61 ± 1.69^c^	39.7 ± 3.53^c,d^
Lbpl	1.95 ± 0.11^a^	29.76 ± 3.76^d^	52.26 ± 1.29^a^	1.79 ± 0.02^a^	28.36 ± 1.54^2,c^	26.7 ± 1.03^b,c^
Lbs1	2.21 ± 0.07^b^	23.67 ± 0.20^d^	56.94 ± 1.79^c,d^	2.19 ± 0.06^d^	42.19 ± 2.31^d^	35.1 ± 1.85^c^
Lbs2	2.23 ± 0.10^b^	7.23 ± 0.53^a^	59.46 ± 0.67^d^	2.28 ± 0.06^e^	19.01 ± 1.24^a^	43.4 ± 1.68^e^
CS	2.13 ± 0.06^b^	19.09 ± 1.71^d^	55.68 ± 0.37^b,c^	1.80 ± 0.03^a^	36.68 ± 3.07^c^	29.4 ± 2.90^b^

pH	Sourdough	Batter before fermentation	Batter after fermentation	Sourdough	Batter before fermentation	Batter after fermentation
Lbf	4.20 ± 0.01^c,d^	4.88 ± 0.10^b^	4.85 ± 0.01^d^	4.05 ± 0.05^c^	4.91 ± 0.04^b^	4.91 ± 0.23^b^
Lbh	4.09 ± 0.01^b,c,d^	4.88 ± 0.02^b^	4.72 ± 0.01^c,d^	3.67 ± 0.01^a,b^	4.32 ± 0.01^a^	4.21 ± 0.01^a^
Lbpa	3.92 ± 0.05^a,b,c^	4.67 ± 0.10^a,b^	4.58 ± 0.09^b,c^	3.71 ± 0.11^b^	4.20 ± 0.30^a^	4.30 ± 0.00^a^
Lbpe	3.76 ± 0.00^a^	4.44 ± 0.01^a^	4.37 ± 0.01^a^	3.53 ± 0.00^a^	4.27 ± 0.00^a^	4.15 ± 0.01^a^
Lbpl	3.77 ± 0.01^a,b^	4.47 ± 0.01^a^	4.37 ± 0.02^a^	3.53 ± 0.03^a^	4.22 ± 0.04^a^	4.14 ± 0.07^a^
Lbs1	4.89 ± 0.26^e^	5.27 ± 0.24^c^	5.05 ± 0.12^d^	4.77 ± 0.28^d^	5.32 ± 0.32^c,d^	5.14 ± 0.20^c,d^
Lbs2	4.14 ± 0.01^d^	4.88 ± 0.01^b^	4.68 ± 0.00^b,c,d^	5.22 ± 0.02^e^	5.39 ± 0.01^d^	5.29 ± 0.01^d^
CS	3.94 ± 0.00^a,b,c^	4.61 ± 0.01^a^	4.50 ± 0.01^a,b^	4.19 ± 0.00^c^	5.09 ± 0.01^b,c^	4.99 ± 0.05^b,c^

TTA	Sourdough	Batter before fermentation	Batter after fermentation	Sourdough	Batter before fermentation	Batter after fermentation
Lbf	18.87 ± 0.11^b^	14.15 ± 0.29^c^	14.36 ± 0.17^b^	12.54 ± 0.23^c^	9.93 ± 0.12^a,b^	10.97 ± 0.10^a,b^
Lbh	20.40 ± 0.47^c^	15.76 ± 0.26^d^	16.40 ± 0.26^d^	17.80 ± 0.57^f^	11.85 ± 0.43^c^	13.26 ± 0.56^c^
Lbpa	17.53 ± 0.16^a,b^	13.43 ± 0.16^b,c^	14.79 ± 0.04^b^	13.33 ± 0.02^d^	13.30 ± 0.41^d^	13.67 ± 0.30^b,c^
Lbpe	25.69 ± 1.02^d,e^	16.45 ± 0.43^e^	17.16 ± 0.36^e^	18.93 ± 0.11 ^g^	14.09 ± 0.53^e^	15.13 ± 0.68^c,d^
Lbpl	25.85 ± 0.28^e^	16.15 ± 0.43^d,e^	16.89 ± 0.39^e^	16.21 ± 0.15^e^	14.04 ± 0.10^d,e^	13.61 ± 0.19^c^
Lbs1	16.74 ± 0.04^a^	11.01 ± 0.10^a^	8.30 ± 0.26^a^	10.55 ± 0.21^b^	10.41 ± 0.62^b^	11.13 ± 0.16^a,b^
Lbs2	20.16 ± 0.18^c^	13.06 ± 0.50^b^	14.76 ± 0.26^b^	9.14 ± 0.27^a^	9.43 ± 0.42^a^	10.57 ± 0.38^a^
CS	22.71 ± 0.19^d^	13.77 ± 0.29^c^	15.36 ± 0.33^c^	16.67 ± 0.13^e^	14.55 ± 0.39^e^	15.52 ± 0.19^d^

Mean value of at least duplicate determinations ± standard deviation. Values associated with different lower case letters denote significant differences (*p* < 0.05)

Lbs1, *Lb. sanfranciscensis* LMG 16002; Lbf, *Lb. fermentum* LMG 6902; Lbpa, *Lb. paralimentarius* LMG 19152; Lbs2, *Lb. sanfranciscensis* DSM 20663; Lbpe, *Lb. pentosus* LMG 10755; Lbpl, *Lb. plantarum* subsp. *plantarum* LMG 6908; Lbh, *Lb. hammesii* DSM 16381

Overall, millet flour was less suitable for GF baking, as the breads achieved lower specific volumes, higher crumb firmness and lower relative elasticities than for buckwheat flour. Due to its poor baking performance and its adverse effect in texture, millet is commonly blended with other GF flours for the production of leavened GF products [15, 16]. It has been reported that the high ash content of millet can cause a gritty texture in bread, which negatively affects its acceptability [17]. This was also observed in the present study. On the contrary, buckwheat breads displayed a pleasant crumb texture, which might be more acceptable by the consumers compared to millet.

As shown in Table 3, crumb hardness and specific volume of sourdough-breads were greatly affected by the different LAB strains, which have also been observed in earlier studies [18]. Lbf, Lbh, Lbs1 and Lbs2 were able to improve the specific volume of millet breads, compared with the CS. Crumb firmness was reduced by Lbpa, Lbh and Lbs2, while an adverse behavior was shown by Lbs1 and Lbf. All Lb. strains were able to enhance the REL of millet breads, except for Lbpa and Lbpl. As for the chemical properties of the millet sourdough, the pH and TTA ranged from 3.53 ± 0.03 to 5.22 ± 0.02 and 9.14 ± 0.27 to 18.93 ± 0.11 mol/L NaOH, respectively. During the fermentation of the bread batters, Lbs1 acidified the pH the most by 0.18 units, decreasing it from 5.32 ± 0.32 to 5.14 ± 0.20 during fermentation. After the fermentation of the batter, highest TTA values were reached by *Lb. pentosus* (Lbpe) (15.13 ± 0.68 mol/L NaOH) and the CS (15.52 ± 0.19 mol/L NaOH).

On the other hand, the Lb. strains adapted differently towards buckwheat flour. Baking results showed that the specific volume of buckwheat breads remained unaffected by most of the Lb. strains compared with the CS, except for Lbf, Lbpa and Lbpl. Lbpa, Lbs2 and Lbpe decreased crumb firmness in buckwheat breads, while the REL could only be improved by Lbs2, compared with the CS. TTA and pH of the buckwheat sourdough varied between 16.74 ± 0.04 and 25.85 ± 0.28 mol/L NaOH and 3.76 ± 0.00 and 4.89 ± 0.26, respectively. Difference in pH between the initial and final fermentation stage of the batters revealed that Lbs1 and Lbs2 were able to decrease this property the most, while Lbpe achieved highest TTA (17.16 ± 0.39 mol/L NaOH) of the fermented bread batter.

In general, the TTA values matched the behavior seen by the pH for both GF flours. The variation in pH and TTA are mainly attributed to differences in microbial metabolisms and acidification rates of the Lb. strains, the type of acids produced and the buffer capacity of the flours. At the same time, acidic conditions of the batters might have affected the main structure-forming components, such as starch and arabinoxylans, altering the quality of the GF breads. Protein solubility was probably increased, influencing the batter matrix and leading to a softer crumb texture.

**Fig. 1:**
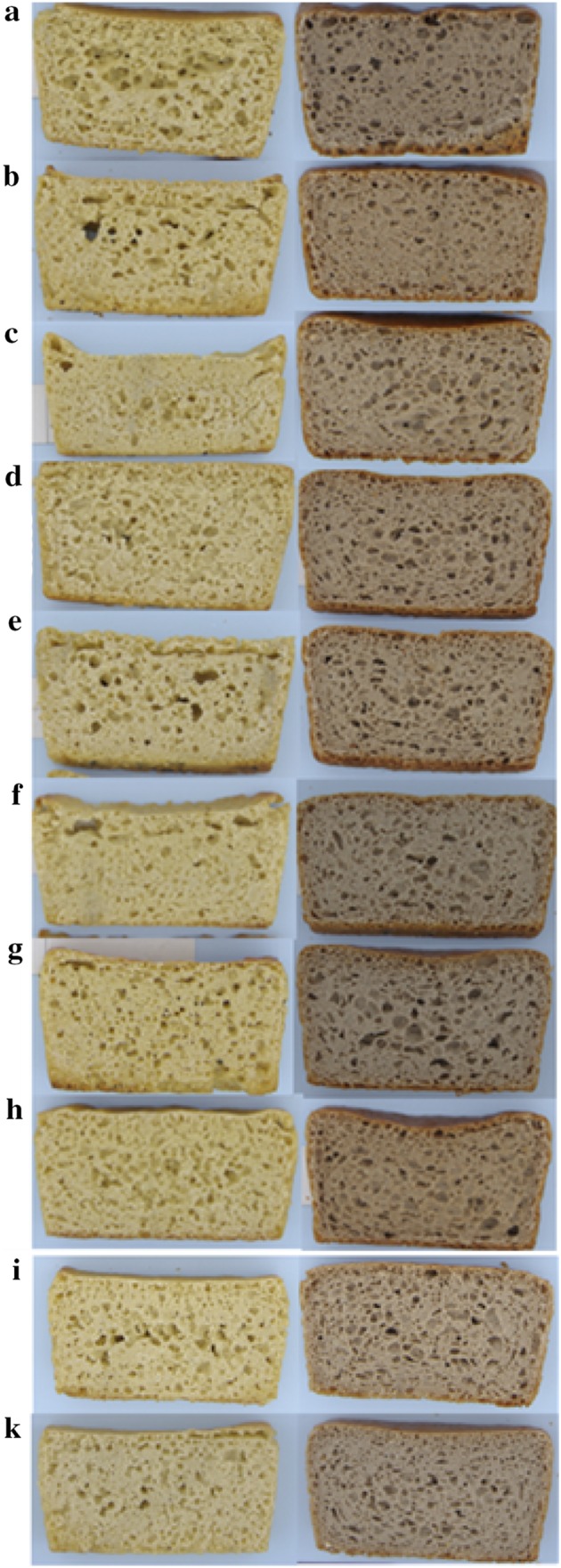
Slices of bread made from whole meal millet (left) and buckwheat (right) flour using a sourdough inoculated with **a** Lbs2, **b** Lbf, **c** Lbpa, **d** Lbs1 **e** Lbpe, **f** Lbpl, **g** Lbh, **h** commercial starter culture or Lbpa, Lbs2, Lbpe and Lbh after **i** day 1, **k** day 10 of back-slopping

Figure 1a–h display the bread slices made from millet and buckwheat flour inoculated with the seven Lb. strains compared with the CS. Differences in crumb porosity are recognizable. Porosity was satisfying in most of the buckwheat breads, while in millet sourdough-breads, dissimilarities in structure and pores were evident.

Interestingly, Lbs1 and Lbs2 showed different adaptability to the flours used, as they affected the bread properties, especially the crumb firmness, in an opposite manner. Previous studies have already reported that when a specific type of exopolysaccharide (EPS) is produced, its molecular size and functional properties can vary according to the conditions (i.e. pH, temperature and sucrose concentration of the media) to which the Lb. strain is exposed to [19, 20]. Since the pH of the sourdoughs produced by Lbs1 and Lbs2 and the crumb firmness of the resulting breads significantly differed amongst each other, it can be inferred that EPS with other functional properties might have caused these variabilities. Another explanation to these discrepancies could be the difference in optimal fermentation temperature (28 °C for Lbs2 compared with 30 °C for Lbs1) and fermentation profiles of the lactobacilli, as reported before by Sohngen et al. [21].

Another factor that could have affected the final bread quality was the source of isolation of the lactobacilli. Considering that Lbf and Lbpl were isolated from fermented vegetables, this factor could have led to an inferior adaption to the different flours and, therefore, to breads with poorest technological properties. Other properties of the sourdough such as presence of yeasts, contaminating microorganisms, bacteriocins or phages could have strongly influenced the bread properties as well. These factors might explain why Lbs1, although isolated from sourdough, still produced breads with inferior properties than other Lb. strains.

Lactobacilli strains, which produced breads with the highest qualitative parameters, were selected as a combined starter for the second stage of the study. The pH of the batter was also a decisive factor for the selection of the strains, as an acidic pH will play an important role in future applied studies and was, therefore, favored. Overall, Lbs2 produced millet and buckwheat breads with the best functional properties, and thus was chosen for back-slopping trials. Lbpe and Lbh influenced positively the crumb firmness of buckwheat and millet bread, respectively, while Lbpa improved this property in both flours. Additionally, these three strains showed a well-suited pH and were also selected for back-slopping.

### Competitiveness of LAB upon back-slopping in buckwheat and millet sourdough

**Fig. 2:**
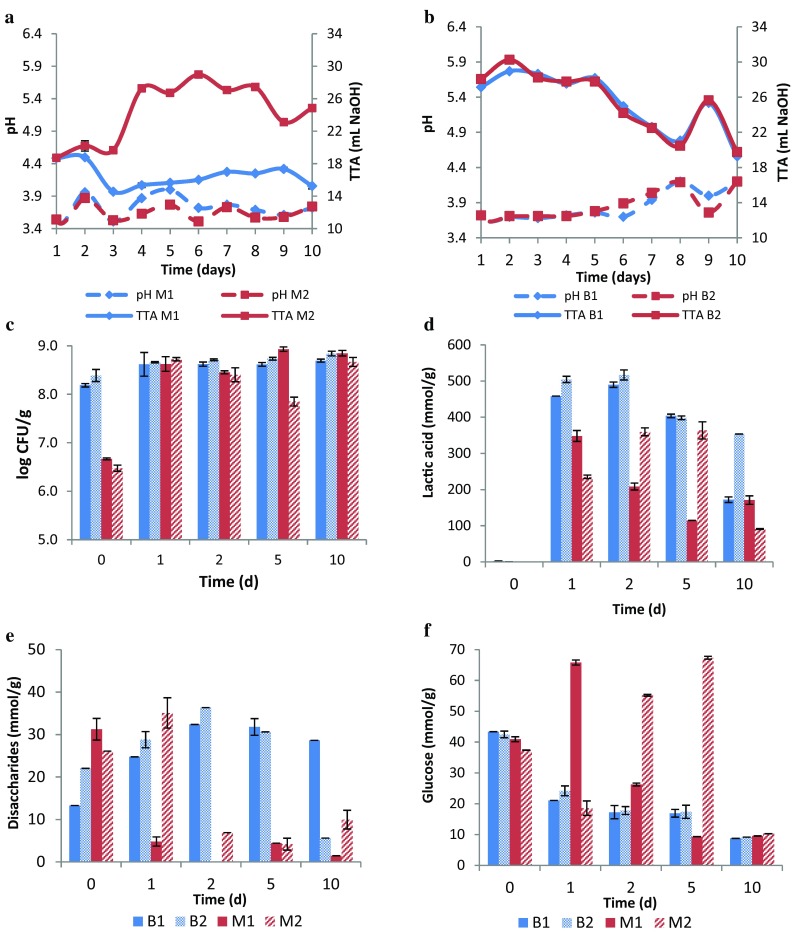
Variation of **a** pH and TTA of M1 and M2, **b** pH and TTA of B1 and B2 **c** microbial count; **d** lactic acid; **e** dissacharide and **f** glucose concentration in the sourdough during a 10 day back-slopping period. M1–M2, B1–B2 represent the individual trials of millet and buckwheat sourdoughs, respectively

Results of the chemical and microbiological properties the millet and buckwheat sourdoughs inoculated with the selected Lb. strains, monitored during a 10-day back-slopping period are presented in Fig. 2. Duplicate measurements were shown separately since some of the trials were not reproducible. The pH of the sourdoughs slightly varied within the testing interval; however, it remained almost constant with increasing number of propagation steps (Fig. 2a, b). The TTA of the buckwheat sourdough tended to decrease with back-slopping, while a non-reproducible trend was displayed by the millet sourdough. The first sourdough batch (M1) showed a decrease in TTA after the second refreshment and remained constant until day 10. In contrast, the acidification rate significantly increased in the second batch (M2) after the third refreshment and thereafter fluctuated moderately. This result was reflected by the microbial count, lactic acid production and the glucose/disaccharide concentration found in the sourdough, being most evident at day 5, where dissimilarities between M1 and M2 were highest (Fig. 2c–f). Microbiological counts ranged from 8.19 to 8.84 log CFU LAB/g in sourdoughs B1 and B2. Initially, M1 and M2 exhibited significantly lower population sizes (6.47–6.67 log CFU/g), which then reached a microbiological count of 7.85 to 8.93 log CFU LAB/g during the refreshment stage. Colony counts of M2 varied to a greater extent during back-slopping compared with the other sourdoughs. It is believed that the difference in microbial stability and carbohydrate metabolization caused different acidification rates of the millet sourdoughs and did, therefore, not allow a reproducible trial. But sequencing analysis of the sourdough isolates showed that the inoculated *Lactobacillus* spp. strains gained in fact dominance over the microbiota of the sourdough during back-slopping (data not shown).

As for the metabolite concentration found in the sourdoughs (see Fig. 2d–f), a higher lactic acid amount was produced in the buckwheat sourdough, which tended to decrease with the number of propagation steps. The disaccharide concentration of the buckwheat sourdough varied from 5.64 ± 0.01 to 36.35 ± 1.92 mmol/g, while the glucose concentration decreased with back-slopping. Regarding the sourdough made with millet, disaccharide and glucose concentration of M1 and M2 significantly fluctuated, showing no specific tendency. This behavior was also seen in the lactic acid concentration, exhibiting an opposing behavior between M1 and M2. In general, M2 seemed to be more unstable than M1, as all of the sourdough properties displayed a more irregular behavior, possibly due to existing contamination microbiota.

Acetic acid could not be detected in most of the sourdoughs. This possibly depended on the degree of aeration of the sourdough, as anaerobic conditions disfavor the production of acetic acid [22]. Only at day 10, a concentration of 12.96 ± 0.88, 50.32 ± 1.51 and 120.91 ± 9.71 mmol/g of acetic acid were found in B1, B2 and M2, respectively. No acetic acid was detected in M1. This acid is often desired when it is present at low concentrations due to its positive contribution to taste. Higher amounts could have detrimental effects on the crumb texture [23].

Overall, the diversity and stability of sourdough microbiota depended on several parameters, which are not fully controllable. The instability of sourdough ecosystems has already been highlighted before. Siragusa et al. [24] assessed the stability of nine strains of *Lb. sanfranciscensis* over a back-slopping period of 10 days, showing that only one autochthonous strain was able to persist during propagation. Brandt et al. [25] reported that a stable microbiota is only achieved when the ratio of the cell density at the beginning and end of the fermentation stage of the different microorganisms, also called multiplication factors, are very similar. Evidently, a constant multiplication factor was not achieved in the millet sourdough and this significantly influenced later results.

### Effect of sourdough maturity on functional bread properties

In general, the selection of the four Lb. strains produced breads with acceptable functional properties and represented a good alternative to commercial starter cultures, as these microorganisms seem to adapt well to the raw materials when added in combination.

Using sourdoughs at different stages during back-slopping (*t* = 1 days; *t* = 10 days), the influence of sourdough maturity on the functional properties of millet and buckwheat breads was evaluated (Table 4). Each bread was baked using the sourdough M1, M2, B1 or B2 from the initial (*t* = 1 days) and final (*t* = 10 days) refreshment stage. As presented in Table 4, the number of propagation steps significantly influenced the functional properties of buckwheat breads. The combined Lb. strains produced buckwheat breads with superior functional properties, as compared with the application of the individual Lb. strains. In regard to millet breads, there was only a significant decrease in crumb hardness after using mature sourdough (*t* = 10 days) but the specific volume and relative elasticity remained unchanged. All functional properties of the millet sourdough-breads showed a higher standard deviation when using the sourdough from the final back-slopping stage. Since the maturity of the sourdough in millet was not reached after the 10-day refreshment period (see Fig. 2), higher differences in bread properties were expected, compared with the buckwheat sourdough, which quickly gained stability upon back-slopping.

**Table 4:** Effect of back-slopping time of millet and buckwheat sourdoughs inoculated with *Lb. sanfranciscensis* DSM 20663, *Lb. paralimentarius LMG 19152, Lb. pentosus* LMG 10755 and *Lb. hammesii* DSM 16381 on the functional properties of sourdough breads

Property^a^	Buckwheat		Millet	
	Day 1	Day 10	Day 1	Day 10
Specific volume (cm^3^/g)	2.46 ± 0.04^b^	2.33 ± 0.03^a^	2.22 ± 0.06^a^	2.14 ± 0.11^a^
Firmness (*N*)	18.12 ± 1.34^b^	6.04 ± 0.21^a^	41.14 ± 3.83^b^	32.21 ± 6.40^a^
REL (%)	52.56 ± 2.66^a^	60.71 ± 1.53^b^	37.58 ± 2.26^a^	38.03 ± 2.43^a^

Mean value of duplicate determinations ± standard deviation. Values associated with different lower case letters for each type of flour denote significant differences (*p* < 0.05) in time

^a^Mean value of functional bread properties made from sourdough B1 and B2 for buckwheat or M1 and M2 for millet, respectively

Figure 1i–k shows the slices of the sourdough breads using the initial and 10-day refreshed sourdough. There was an improvement in the crumb structure after back-slopping, especially in the millet bread, as the slices appear to have a more homogeneous pore structure.

## Conclusion

This investigation was able to evaluate the performances of different *Lactobacillus* spp. strains applied in the fermentation of millet and buckwheat sourdoughs. Results demonstrated that the combination of Lbs2, Lbpa, Lbpe and Lbh could be used as potential starter cultures for millet or buckwheat sourdough-breads. Since the maturity of the millet sourdough was not reached in such a short back-slopping period, an extended refreshment interval should be carried out to ensure constant bread quality.

This study was able to show that sourdough technology alone is able to improve the quality of GF breads when using the appropriate lactobacilli strains. This could meet the consumers demand for clean labeling, natural products and a reduced use of food additives [8].
